# Evidence for the Identification of Carabayo, the Language of an Uncontacted People of the Colombian Amazon, as Belonging to the Tikuna-Yurí Linguistic Family

**DOI:** 10.1371/journal.pone.0094814

**Published:** 2014-04-16

**Authors:** Frank Seifart, Juan Alvaro Echeverri

**Affiliations:** 1 Max Planck Institute for Evolutionary Anthropology, Leipzig, Germany; 2 Universidad Nacional de Colombia, Amazonia Campus, Leticia, Colombia; Stony Brook University, United States of America

## Abstract

This paper provides evidence for the identification of the language of the uncontacted indigenous group called Carabayo, who live in voluntary isolation in the Colombian Amazon region. The only linguistic data available from this group is a set of about 50 words, most of them without reliable translations, that were collected in 1969 during a brief encounter with one Carabayo family. We compare this material with various languages (once) spoken in the region, showing that four attested Carabayo forms (a first person singular prefix and words for ‘warm’, ‘father’, and ‘boy’) display striking similarities with Yurí and at least 13 Carabayo forms display clear correspondences with contemporary Tikuna. Tikuna and Yurí are the only two known members of the Tikuna-Yurí linguistic family. Yurí was documented in the 19th century but has been thought to have become extinct since. We conclude that the Carabayo – directly or indirectly – descend from the Yurí people whose language and customs were described by explorers in the 19th century, before they took up voluntary isolation, escaping atrocities during the rubber boom in the early 20th century.

## Introduction

There are still around 100 uncontacted indigenous groups around the world, and a few dozen of them in the Amazonian rainforest, according to the NGO Survival International (http://www.survivalinternational.org/tribes). Most of these groups are known to be closely related linguistically and culturally to groups already contacted. However, not much more than their mere existence is known about some of them. This is the case for the Carabayo people who live in the remote upper River Puré and River Bernardo region in the Colombian Amazon rainforest. The name Carabayo derives from the (mock) name “Bernardo Caraballo”, which was given to a Carabayo man by local people during a brief encounter in the Colombian town La Pedrera (Bernardo Caraballo was the name of a Colombian boxing champion). Subsequently the Carabayo people and their language have been referred to as Caraballo [1], [2]. The 2013 *Ethnologue* language catalogue [3] introduced Carabayo as an English version of the language name, and assigned the ISO 639-3 code cby to it.

In the current study, we analyze the only linguistic data available from this group, around 50 words that were overheard and noted down during a brief encounter with one Carabayo family in 1969, showing that the Carabayo most likely speak a language closely related to Yurí (also spelled Jurí) as well as to Tikuna, which have previously been shown to be related to each other [2], [4]–[7]. The Yurí language was documented in four wordlists in the 19th century but had been presumed to have become extinct since. Tikuna is still spoken by about 40,000 speakers, mostly along the Amazon River in Peru, Colombia, and Brazil. If Carabayo is relatively closely related to both of these languages, as we suggest here, one possibility would be that it may be part of a former dialect continuum circumscribed by the Tikuna and Yurí languages.

Our study substantiates previous claims of a link between Carabayo and Yurí that were either based on limited data and non-rigorous methods [8] or did not substantiate this claim with linguistic data at all [9], [10]. We also substantiate the existence of similarities between Carabayo and Tikuna that were noted by Goulard & Montes Rodriguez [2] based on incomplete Carabayo materials which they considered to be too poor to draw any further conclusions. Our identification is based on a comparison of all available Carabayo data (from three different sources) with, firstly, four Yurí wordlists collected in the early to mid-19th century, one of which has only recently become accessible [11]: Natterer's Yurí wordlist was thought to have been destroyed in a fire in Vienna in 1848, until it was discovered in the late 1970s by Ferdinand Anders in the University Library of Basle. The handwritten manuscript has recently been transliterated by Hélène B. Brijnen at Leiden University. (Incidentally, the Carabayo wordlist [12] was also not accessible [13] until recently, because the Capuchin missionary publication *Amanecer Amazónico*, in which it was published, was not distributed widely. Additionally, the issue of *Amanecer Amazónico* that contains the Yurí wordlist is missing in both the Capuchin missionary archive in Leticia and in the national library of Colombia, the *Biblioteca Luis Ángel Arango* in Bogotá. It was eventually located by the first author in the *Arxiu Provincial dels Caputxins de Catalunya* in Sarrià, Barcelona.) Secondly, we compare Carabayo with contemporary Tikuna data provided by a native speaker of Tikuna. Our identification of the relationship of Carabayo with Yurí and Tikuna also implies that Tikuna should no longer be considered a language isolate with no living relatives [3].

The nature and scarcity of the available Carabayo data implies that standard methods for identifying languages – e.g. by frequent sequences of sounds or letters [14] – or for establishing genealogical relations between languages – e.g. by regular sound changes in sets of cognate words [15] –cannot be applied straightforwardly. Our analysis of the available Carabayo data thus draws on a variety of methods to derive evidence for the likelihood of an identification of Carabayo. These include phoneme frequency counts, semantic extensions of words, taking into account the context in which Carabayo words occurred, morphological composition of words, and the relative borrowability of different sections of vocabulary.

The Carabayo material investigated here was collected in 1969 from people who live in the upper River Puré/upper River Bernardo area, between the Putumayo and Caquetá rivers in the Colombian Amazon region [16]. In early 1969, a local Colombian and a local Miraña Indian undertook an expedition to the Carabayo's territory. When they did not return, a military commission that was sent to rescue them made violent contact with the Carabayo people and took one family hostage. This family, consisting of an adult couple and three children, was then held in the boarding school of the Capuchin mission in the Colombian town La Pedrera for a few weeks before they were ‘repatriated’. During this encounter, the Carabayo data analyzed here were collected. Since then, Carabayo people and traces of them have been sighted on various occasions, primarily by members of the cocaine mafia and guerilla fighters [16]. The most recent evidence of the Carabayo's persistence are aerial photographs of their roundhouses taken in 2010 [16].

We strongly disapprove of the circumstances under which Carabayo data were collected. We hope that our study of the Carabayo material that was published in reports of these dire events contributes to the protection of the Carabayo people, in line with, for example, Survival International's policy of making knowledge about uncontacted peoples public in order to raise awareness of the threats they are facing. In 2002, the Río Puré National Park was created to protect the Carabayo's territory. In addition, a legal decree passed in 2011 guarantees uncontacted peoples in Colombia such as the Carabayo the rights to their voluntary isolation, their traditional territories, and reparations if they face violence from outsiders.

Since the language of the Carabayo was unintelligible to any of the indigenous peoples present in La Pedrera in 1969, it is a mystery as to who the Carabayo are. Trupp [9] and Landaburu [10] have hypothesized that the Carabayo people might be descendants of the Yurí, without, however, discussing linguistic evidence [13]. They were apparently not aware of Vidal y Pinell's [8] attempt to analyze the Carabayo data published by Juan Berchmans de Felanix [17]. (Note that names of Capuchin monks are composed of a religious name followed by the place of their origin and that they are ordered alphabetically by the religious name, following the conventions established by the *Lexicon Capuccinum* [18]. The secular name of Juan Berchmans de Felanix was Antonio Font.) Vidal y Pinell concluded that the language of the Carabayo corresponds to Yurí, based on two arguments. Firstly, he suggests that three items from a Yurí wordlist by Wallace [19] correspond to items collected from Carabayo in 1969 by Juan Berchmans de Felanix [17]. Secondly, Vidal y Pinell [8] compared the frequencies of the sound *t
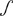
*, which he considered the most “representative” phoneme in Wallace's Yurí data, in Carabayo, Yurí, and various other languages spoken in the region, for which Wallace [19] also provides wordlists. He found that it occurs in 23% of the words in Wallace's Yurí word list (18/77) and in 21% of the words from Juan Berchmans de Felanix's Carabayo list (7/33), but in maximally 8% of the words of Kubeo, Tucano, Kueretú, Tariana, and Baniwa. From these two pieces of evidence, Vidal y Pinell [8] concludes that the Carabayo that were sighted in 1969 are descendants of the Yurí, documented by Wallace around 1850. The current study support the hypotheses of a genealogical link between Carabayo and Yurí, based on a much more detailed discussion of potentially cognate forms. Crucially, this discussion is based not only on Yurí data collected by Wallace but also on Yurí data collected by Spix, Martius, and Natterer. In addition, we include correspondences with contemporary Tikuna in our discussion.

## Materials and Methods

For the purpose of the current study, all attested linguistic material reported for the Carabayo family in 1969 has been gathered (Table 1). Most of it is from the list published by Juan Berchmans de Felanix [17]. A few additional items are interspersed in two descriptions of the encounter with the Carabayo in 1969: One by Juan Berchmans de Felanix himself [20] and another by Venanci d'Arenys de Mar [21]. These items include four clearly Spanish words that the Carabayo reportedly used but that were apparently learned during the brief encounter, i.e. *tabako* (item 1) and *karaβayo to comes* from item 27, which probably correspond to the Spanish-based name given to the Carabayo man by the people of La Pedrera and to Spanish *tu comes* ‘you eat’. Excluding these four words, there are a total of 55 word tokens. Within these, one word occurs twice (*uro*, item 6), two three times (*
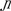
a*, items 3, 7; *kariba*, items 3, 5, 28), and one four times (*ane*, after merging *n* and *nn*, see below, items 29, 30, 35), i.e. there are 48 word types in the data. Two elements in the list are of Nheengatu (Tupian) origin, which was the lingua franca used in the area in the 17 h, 18th and 19th centuries. These are *kariba* ‘white man’ (items 3, 5, 28), and *tupana* ‘God’ (item 23).

**Table 1:** All attested linguistic material for Carabayo,

	Carabayo	gloss, explanation, or context	source
1	tabako	‘tobacco’	[20]
2	hako	at being frightened by dogs; ‘bite’ according to Juan Berchmans de Felanix [17]	[17], [20]
3	′ 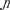 a kariba, ′ 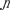 a irobe	shouted at white people by an old women during the occupation of her house. Castro Caycedo [30] reports that Carabayo contacted on a path shouted *kariba, kariba ñé*	[20]
4	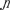 e	‘no’	[17], [21]
5	kariba	‘white man’	[17], [21], [30]
6	uro, uro	when meeting a white man in the bush, pointing at direction opposite to where he came from	[20]
7	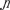 a-nauué	‘give me, show me’	[17]
8	gudda	‘wait’	[17]
9	agó	‘bring’	[17]
10	amá	‘come’ (Spanish *siga*)	[17]
11	ao	how the children call their father	[17]
12	aua	calling a child	[17]
13	gu	‘yes’	[17]
14	hono	‘boy’	[17]
15	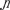 a	‘out’, maybe based on item 03	[17]
16	pama	‘there, look!’	[17]
17	pin 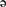	‘shrimp/prawn’	[17]
18	pin 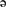 -gó	‘bring shrimp/prawn’ (see items 09, 17)	[17]
19	t 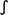 auameni	‘good, well, like’	[17]
20	t 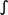 aunoβe	‘warm me!’ (the speaker ordered a child to warm his hands with fire and apply them to his body)	[17]
21	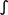 àma	‘enough!’	[17]
22	alo	‘come!’	[17]
23	tupana	‘God’	[21]
24	jakoma	boy's name; according to Bergès [26] the autodenomination of the Carabayo is *yacumo*.	[21]
25	jakomanate	man's name	[21]
26	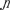 ego	‘child(ren)’, used by Carabayo woman addressing (two of) her children	[21]
27	oro kami karaβayo to comes	‘give me meat, Carabayo wants to eat’	[21]
28	kariba dimene	(during forced walk through jungle), *dimene* means ‘kill’ according to Juan Berchmans de Felanix [17]	[17], [20]
29	ané ui kon 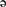	/	[17]
30	ané uikar 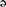	/	[17]
31	ar 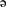 t 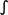 e o neko	/	[17]
32	bajaneku	/	[17]
33	ekoneko	/	[17]
34	en 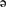 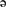 n 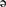 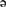 nna pikhu	/	[17]
35	er 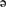 anne anne	/	[17]
36	etamenita	/	[17]
37	βadajareu	/	[17]
38	jua nekon 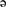	/	[17]
39	nenerigu	/	[17]
40	t 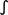 auiba t 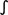 utaiba	/	[17]

Juan Berchmans de Felanix [17] notes that the translations he provides are very hypothetical, in fact mere guesses, given that he and the Carabayo had no common language. Juan Berchmans de Felanix was a native speaker of Catalan and fluent in Spanish. His publication with the Carabayo vocabulary was written in Spanish and meant for a Spanish-speaking readership. We thus assume that the phonetic values of the consonants and vowels in his representation of Carabayo correspond to those of Spanish graphemes and the Carabayo material was transliterated to IPA symbols accordingly. Additionally, *x* (item 21) was transliterated as *
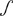
*, following the pronunciation rules of Catalan, Juan Berchmans de Felanix's native language, since [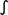] has no graphic representation in Spanish. We transliterated *ê* as *
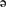
* based on his remark that it stands for “ê neutra francesa [neutral French ê]” [17]. An acute accent appears in only four items (7, 9 = 18, 10, and 29 = 30). This suggests that whatever it may represent, it was not systematically marked. Therefore we disregard it for establishing hypothetical phoneme inventory.

Tables 2 and 3 are hypothetical phoneme charts of Carabayo with indications of phoneme frequencies in the extant data. Some aspects of this hypothetical phonology must remain uncertain because a few putative phonemes occur only once or twice (in parentheses in Tables 2–3). Therefore, it is doubtful whether geminates, aspirated consonants, and long vowels really exist in Carabayo. Note that the absence of *s* is confirmed by Juan Berchmans de Felanix's [17] observation that the Carabayo man pronounced Spanish very well, except for *s*, which he pronounced as *
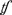
*. Despite the scarcity of the data, Tables 2 and 3 represent what might be a perfectly plausible and also typically Amazonian phoneme system, suggesting that a comparative analysis can reasonably be carried out with these data.

**Table 2:** Carabayo consonants.

	bilabial		alveolar		palatal		velar		glottal
**plosive**	b_6_	p_5_	(d_2_)	t_5_			g_6_	k_13_	
**(plosive geminate)**			(dd_1_)						
**(plosive aspirated)**								(kh_1_)	
**fricative**	(β_2_)				j_5_			(x_1_)	(h_2_)
**affricate**						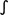 _5_			
**approximant**									
**nasal**	m_9_		n_22_		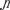 _6_				
**nasal geminate**			nn_3_						
**Flap**			r_11_						
**(liquid)**			(l_1_)						

Subscript numbers represent frequency of occurrence of the phoneme in the corpus (phonemes in parentheses occur only once or twice).

**Table 3:** Carabayo vowels.

i_15_	e_26_	o_22_
u_17_/(uu_1_)	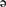 _7_/( 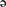 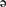 _2_)	a_52_

Subscript numbers represent frequency of occurrence of the phoneme in the corpus (phonemes in parentheses occur only once or twice)

Can we tell from this material whether the Carabayo language is related in any way to any other known language? One hypothesis would be that they speak a closely related variant of a living language. This appears to be the case in neighboring Peru, where most uncontacted groups are linguistically related to groups already contacted, which allows one to have some degree of previous knowledge of their language. However, in the case of the Carabayo, this is unlikely because, while the Carabayo family was held at La Pedrera, speakers of the following languages were asked to try to communicate with them, without success [8], [20] (language names are given in standard spelling and with genealogical affiliation and ISO 639-3 codes): Andoke (isolate, ano), Muinane (Boran; bmr), Witoto (Witotoan; three varieties: Mηnηca hto; Murui huu; Nüpode hux), Mirañas and Boras (Boran; both boa), Carijona (Cariban; cbd), Yucuna and Matapí (Arawakan; both ycn), Tanimuca (Tucanoan; tnc), Cabiyarí (Arawakan; cbb), Tikuna (Tikuna-Yurí; tca), Ocaina (Witotoan; oca), Nonuya (Witotoan; noj), Puinave (isolate; pui), Tukano (Tucanoan; tuo), Yuhup (Nadahup; yab). These include all living languages spoken in the region where the Yurí were sighted, and we can thus discard this hypothesis (but see our discussion on Tikuna below).

A second hypothesis is that Carabayo corresponds or is closely related to an extinct but documented language of the region. Based on information given by Bartolomé de Igualada & Marcelino de Castellví [22] and Marcelino de Castellví & Espinosa Pérez [23], as well as other historical sources summarized by Franco [16], the languages given in Table 4 are possible candidates for identifying Carabayo since they were reportedly spoken in the same area, some of them up until the early 20th century. All of them were documented in wordlists in the first half of the 19th century, and all of them are presumed to now be extinct (although Mura's dialect – or sister language – Pirahã survives). Three closely related Arawakan languages [24] were reportedly spoken in the region (Uainuma, Jumana, Passé). Among these, Passé and Uainuma were chosen for the comparison since they are the ones geographically closest to where Carabayo were last sighted.

**Table 4:** Candidate languages for the identification of Carabayo.

Language	Affiliation	Evidence for affiliation
Coëruna	possibly Witotoan	Koch-Grünberg [31], Loukotka [32]
Coretú	Tucanoan	Loukotka [32]
Mura	Mura(-Pirahã)	Campbell & Grondona [33]
Passé	Arawakan	Ramirez [24]
Uainuma	Arawakan	Ramirez [24]
Yurí	Tikuna-Yurí	Carvalho [7], Goulard & Montes Rodriguez [2]

All of the wordlists for the languages listed in Table 4 were published by Martius [25], except for Natterer's [11] Yurí wordlist (see below). The wordlists were collected in 1819 and 1820 by the German botanists Carl Friedrich Philipp von Martius (Coretú, Coëruna, Mura) and Johann Baptist von Spix (Passé). The Yurí and Uainuma wordlists combine words collected by Martius and by Spix as well as words collected by Alfred Russell Wallace around 1850, which Wallace himself also published in an appendix to Wallace [19]. The Austrian naturalist Johann Natterer collected an additional wordlist on Yurí in 1833 [11]. These wordlists cover basic vocabulary and local fauna and flora terms.

Finally, it is also possible that the Carabayo speak a language that has never been documented. In this context it is noteworthy that a number of languages of the area were documented for the first time as late as the early 20th century, among them Ocaina, Nonuya, and Resígaro, showing that some languages remained unnoticed for a long time after the region had begun to be explored. However, during the 1930s, the indigenous groups of the Caquetá-Putumayo area of the Colombian Amazon region were surveyed in great detail by Capuchin missionaries, including the Ocainas, Nonuyas, and Resígaros [22], [23]. Based on information from these surveys, Marcelino de Castellví and Espinosa Pérez [23] suggest that Yurí speakers persist in locations very close to where the Carabayo were sighted in 1969, without, however, giving linguistic data as evidence.

## Results and Discussion

Our comparison of the Carabayo data with Coëruna, Coretú, Mura, Passé, Uainuma, and Yurí revealed that a number of Carabayo forms match corresponding Yurí elements, but none match forms of the other languages. Among the Carabayo-Yurí correspondences is one that Vidal y Pinell [8] had identified, Carabayo *ao* ‘father’, as we discuss below. The other two Carabayo-Yurí correspondences given by Vidal y Pinell [8] do not hold up to scrutiny: He suggests that Carabayo *aua*, which according to Juan Berchmans de Felanix [17] might mean ‘child’, corresponds to Wallace's Yurí *owúye* ‘son’. This correspondence seems far-fetched and cannot be confirmed by other Yurí forms such as *o nné* ‘son’, *o e˜n* ‘child’, *ta ünna* ‘boy’ (Natterer), *oná* ‘son’, *uhé* ‘child’ (Martius), or *suuné* (Spix). (Incidentally, Wallace's *owúye* ‘son’ probably means ‘daughter’, rather than ‘son’, as the forms for ‘daughter’ given by the three other sources for Yurí suggest: *zo åbü* (Natterer), *tschöwü* (Martius), *suabüe* (Spix). The first syllable in these three is the first person possessor marker.) Furthermore Vidal y Pinell [8] suggests that the Carabayo form *ñe*, reported to mean ‘no’, corresponds to Wallace's Yurí *eeñ* ‘bad’. Again, this seems far-fetched and cannot be confirmed by other Yurí forms for ‘no’: *ka* (Natterer), *tiwá* (Martius), *ghainà* (Spix).

There are a number of other forms, however, that display intriguing correspondences between Carabayo and Yurí and that were not detected by Vidal y Pinell [8], partially because he did not have access to Martius', Spix's, and Natterer's Yurí data. The relevant Carabayo and Yurí data are presented in Table 5.

**Table 5:** Summary of Carabayo and Yurí data compared.

	Carabayo	Yurí material compared
1	*t 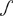 aunoβe* ‘warm me’	*t 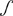 au-* + *noré* ‘warm’ (Wallace)/*tso tsórerú* ‘warm’ (Natterer)
2	*t 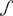 auameni* ‘good, well, like’	*t 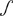 au*- + *(su-)mêniko* ‘(my) heart’ (Spix)
3	*t 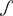 auiba*	*t 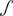 au*- + *(tschu-)ibaüh* ‘(my) back’ (Martius)
4	*t 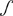 utaiba*	*t 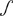 au*- + *taobi* (Martius) ‘body’/*taiaeboí* (Martius) *toipuy* (Spix) ‘week’/*taiaeboí* (Martius), *toipuy* (Spix), *tai ro˜n i* (Natterer) ‘night’
5	*ar 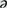 t 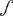 e o neko*	*a aré* (Natterer), *áhre* (Martius), *aré* (Spix), *ahri* (Wallace) ‘red’ + *tschauúnäco* ‘I bury’ (Martius)/*t 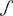 au* + *nihcó* ‘live’ (Martius)/*tschu*-*inicko* (Martius), *subinigho* (Spix) ‘my testicles’
6	*hono* ‘boy’	*o nné* ‘son’, *o e˜n* ‘child’, *ta ünna* ‘boy’ (Natterer), *oná* ‘son’ (Martius), *(su)uné* ‘(my) son’ (Spix)
7	*ao, -nate* ‘father’	*atú* (Natterer), *hato* (Martius), *háto* (Wallace), *(su)âtu* ‘(my) father’ (Spix)
8	*hako* ‘well!’	*hokó* ‘I am fine, this is good’ (Natterer), *ockó* (Martius) *ukó* (Spix) ‘beautiful’

Item 1 in Table 5 contains a complex form in which probably both elements correspond. The first element, *t
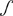
au-* is well attested in Yurí as a first person subject and possessor prefix. It appears in various spellings in Yurí data, e.g. *tschau-, tschu-* (Martius), *su-* (Spix), and *tcho-* (Wallace). The apparent mismatch between first person subject form and second person reference in item 1 could easily have arisen due to the lack of a common language in the situation in which the form was noted by Juan Berchmans de Felanix [17]: It is common even in professional fieldwork elicitation situations that, for example, in response to a field worker asking for a translation of “I sit”, an informant provides a form meaning “you sit”, referring to the field worker. Alternatively, *t
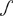
au-* in item 1 may be an object pronoun followed by a prefixless imperative verb form in item 1. The second element, *noβe* ‘warm’ matches well with Wallace's Yurí *noré* ‘warm’. It matches less well with Natterer's form for ‘warm’, but within this form *ore* is shared. Item 2 in Table 5 is a less clear case, but it might be argued that a first person singular form is likely to occur in a form translated as ‘like’. The correspondences involving Carabayo *t
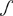
au-*, *t
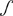
u-*, and *t
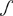
e* proposed in items 3–5 in Table 5 are more hypothetical since no information on their meaning in Carabayo is available. However, they might contain further instances of the word-initial first person singular prefix. In Yurí, variants of *t
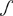
au-*, probably conditioned by the stem to which it is prefixed, are attested, primarily *t
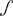
u-*, e.g. *tschu-báacki* ‘my elbow’ (Martius). The Carabayo words beginning with *t
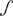
au-*, *t
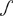
u-*, and *t
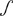
e* given in items 3–5 might thus well be nouns with a first person singular possessor prefix or verbs with a first person singular subject prefix that Juan Berchmans de Felanix [17] overheard from the conversations among the Carabayo. Note that the occurrence of *t
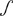
au-*, *t
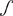
u-* is also responsible for the high frequency of *t
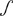
* in both Carabayo and Yurí, which Vidal y Pinell [8] noted.

Item 6 in Table 5, *hono* ‘boy’, constitutes a reasonably certain correspondence in terms of a sequence of a back rounded vowel (*o* or *u*) followed by *n* and possibly another, unidentified vowel, and is attested as such five times in the Yurí data, including attestations from three different sources. Item 7, Carabayo *ao* ‘father’, also matches reasonably well with Yurí data, as already noted by Vidal y Pinell [8], in terms of the initial vowel *a* and final vowel *o*, which alternates with *u* in the Yurí data. A form related to Yurí *(h)ato, atu* ‘father’ may also be identifiable in Carabayo *jakomanate*, the Carabayo man's name, when compared with *jakoma*, the Carabayo man's eldest son's name, according to Venanci d'Arenys de Mar [21] (items 24 and 25 in Table 1). If one assumes that the first term literally means ‘Jakoma's father’, then *nate* would mean ‘father’. This form matches attested Yurí forms relatively well, and it is strikingly similar to Tikuna (Yurí's sister language) *nat
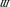
* ‘father’. The use of teknonyms is not attested for Tikuna or for other indigenous groups in the direct vicinity, but it is attested in other places in Amazonia. In any case, it seems clear that *jakomanate* is a complex form and it is likely that *-nate* means ‘father’, even if the Carabayo do not employ a conventionalized system of teknonyms. Note that Venancy d'Arenys de Mar [21] claims that the Carabayo man called *jakomanate* was not the father of the oldest Carabayo boy, who was called *jakoma*, but maybe his brother, without, however, providing any evidence or further argumentation for this claim. This claim contradicts all other sources, who assume they were father and son, especially Bergès [26], who probably knew the Carabayo best. Even if they were not father and son, they may have used a teknonym since it has been observed elsewhere in Amazonia that teknonyms are applied among relatives or people living together [27].

The Carabayo expression in item 8 in Table 5 is translated in Juan Berchmans de Felanix [17] as ‘bites’. However, the context where this word was recorded is described by Juan Berchmans de Felanix [20] as follows: Shortly after the Carabayo family was captured, they were led, bound, through the jungle. When they arrived at a place where the commission had left their dogs behind, the Carabayo family showed fear and repeated various times the word *hako* (“Al llegar al sitio donde estaban los perros, demostraron miedo, repitiendo distintas veces la palabra JACO” [20]). In this context it is possible that *hako* is some kind of interjection, especially because it was repeated various times. If so, it matches well with the Yurí form *hokó* which is given by Natterer as an equivalent of both ‘this is good’ [German *dies ist gut*] (contrasting with ‘this is not good’ [German *dies taugt nichts*], the preceding entry in Natterer's list) and ‘I am fine’ [German *Mir geht es gut*] (apparently as an answer to ‘how are you?’ [German *Wie geht es dir*], the preceding entry in Natterer's list). Natterer's Yurí *hokó* probably corresponds to Martius' Yurí *ockó* and Spix's Yurí *ukó*, both given as equivalents of ‘beautiful’ [Latin *pulcher* in the original list]. The fact that it appears in various contexts suggests that Yurí *hokó* is a more widely applicable expression that may be translated as “well” and that can also be used as an interjection rather than a literal translation of the equivalents given by Martius, Spix, and Natterer. Our experience with indigenous people of the area suggests that it is not unlikely that the same expression would be used in the contexts described for Carabayo *hako* as well as in the ones described for Yurí *hokó, ockó*, and *ukó*. For instance, the Bora people, the Yurí's neighbors to the west, would use *tehdújuco*, which literally means ‘already like this’, in all of these contexts.

Additionally, we note that there are a number of further, far more hypothetical correspondences contained in the data summarized in Table 5. Firstly, Carabayo *meni* (item 2) may correspond to Yurí *meniko* ‘heart’ if one assumes that an expression translated as ‘good, well, like’ would be expressed as ‘(pleases) my heart’. Furthermore, in the Carabayo material for which no translation at all is provided, a number of forms can be identified that match Yurí forms, as noted in Table 5. For instance, *ar
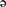
* in item 5 may correspond to Yurí *are* ‘red’, which is well attested in various sources for Yurí.

The two Nheengatu elements in Carabayo, *kariba* ‘white man’ and *tupana* ‘God’ are also attested in Yurí data: *kalibåå* (Natterer) and *tupana* (Martius). These correspondences do not provide evidence for an identification of Carabayo with Yurí because both items are widespread among languages of the region. However, the exact match between Yurí *tupana* (Martius) and Carabayo *tupana* is noteworthy, given that this form was apparently phonologically nativized differently in Coeruna, as *toibá*, and in Mura, as *tupaua*. For Uainuma, *tupana* is reported, as well. For the other two candidate languages, words for ‘God’ which are non-related and probably native are documented, i.e. *pokené* for Passé and *nümúpalŭghtără* for Coretú. No forms corresponding to *kariba* are attested in any of the candidate languages, except for Yurí, due to the fact that there were no entries for this concept in the wordlist template that Martius, Spix, and Wallace used.

In summary, we can identify in Carabayo data four forms that match corresponding Yurí forms well: a first person singular prefix, and words for ‘warm’, ‘boy’, and ‘father’, in addition to a more hypothetical correspondence involving an interjection ‘well!’. Table 6 contrasts these Carabayo-Yurí correspondences with non-corresponding forms of other candidate languages.

**Table 6:** Corresponding elements Carabayo-Yurí vs. other candidate languages (NA - no form attested with a comparable meaning).

	Gloss	Carabayo	Yurí	Coeruna	Uainuma	Passé	Coretú	Mura
1	I, my	*t 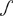 au-, t 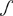 u-*	*t 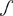 au-, t 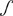 u-*	*kui-, ku-*	*no-, nu-*	*no-, na-*	*yi-*	*tschäng*
2	warm	*noβe*	*nore*	(NA)	*amoiri*	(NA)	(NA)	(NA)
3	boy, son	*hono*	*ona, one*	*quäda*	*-iri*	*aghunghii, tschiker-noma*	*simagö*	*oahabäh*
4	father	*ao*	*(h)ato, atu*	*Domú*	*pai*	*payü*	*tsáackö*	*itohúaeng*
5	well!	*hako*	*hokó*	(NA)	*misare*	(NA)	(NA)	(NA)

The strongest evidence for a link between Carabayo and Yurí comes from the first person singular prefix (item 1 in Table 6). It is attested in one complex Carabayo form, for which there is a matching translation (*t
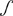
aunoβe* ‘warm me’), and may be contained in further Carabayo forms given by Juan Berchmans de Felanix [17] without translations. For this form, the absence of corresponding forms in other candidate languages is particularly telling since for a first person singular pronoun, the absence of a corresponding form in a candidate language is not likely due to alternative words with similar meanings that happen to be recorded in the extant wordlist (as can easily happen with words for ‘warm’, ‘boy, son’, ‘father’, and ‘well!’). Additionally, the first person singular forms of Yurí, Coeruna, Uainuma, Coretú, and Mura given in Table 6 have etymologies in their linguistic families, which excludes the possibility that they are mistakenly given as first person forms in the wordlist collection in the 19th century. And finally, personal pronouns are known to be especially resistant to borrowing [28], i.e. their similarity is truly indicative of a genealogical link, not of language contact.

It should be noted that any of the suggested correspondences given in Table 6 involve a fair amount of speculation due to the scarcity of information on Carabayo as well as on Yurí. For instance, *t
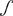
aunoβe* recorded in the context “the speaker ordered a child to warm his hands with fire and apply them to his body,” could mean many other things, e.g. ‘touch me’, ‘rub me’, or ‘hug me’. In addition, there is an unexplained correspondence between the bilabial consonant *β* in Carabayo *noβe* and the alveolar consonant *r* in Yurí *nore* ‘warm’. However, the existence of a whole set of five at least potentially matching forms shared by Carabayo and Yurí, and the lack of any matching forms in other candidate languages does strongly suggest that if Carabayo is related to any of the candidate languages, it is most likely related to Yurí.

Our comparison of Carabayo and Tikuna revealed a high number of very good matches between Tikuna and Carabayo, as summarized in Table 7. The Tikuna correspondences to Carabayo were provided by Abel Antonio Santos Angarita, a native speaker of Tikuna and trained linguist specializing in Tikuna dialectology [29], on inspection of the Carabayo material. These data contain at least 13 close correspondences. Among these, six items (numbers 1–7 in Table 7) constitute very good matches, both semantically and phonologically. Another six items (numbers 8–13 in Table 7) can be considered good matches. Another three items (numbers 14–16) are given here that match less well but are still worth considering (item 14 provides an alternative correspondence for *hako*). What adds credibility to the matches in Table 7 is that they exhibit regular sound correspondences between Carabayo and Tikuna, especially Carabayo *g* (or *k*) – Tikuna *η* and loss of word-initial *n* in Carabayo, both of which are attested in three well-matching pairs (counting also one case of loss of word-initial *
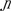
i*). The matching elements include a number of items that are cross-linguistically very hard to borrow, especially first and third person pronouns and the verb ‘come’ [28]. Even for the other items, similarity is unlikely due to contact since there is a strong cultural avoidance of lexical borrowing in the entire region, and it is unlikely that the Carabayo would be an exception.

**Table 7:** Carabayo-Tikuna correspondences (Abbreviations: sg – singular; pl – plural; # - word boundary; Ø – elision).

	Carabayo	Tikuna	Sound correspondences
1	* 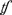 auameni* ‘good, well, like’	* 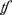 au na me nii* (1sg/3sg/like/be) ‘I like it’ (lit. ‘it is good to me’)	*Ø-#n*
2	*gudda* ‘wait!’	*η 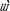 ná* ‘wait!, not yet’	*g-η, dd-n, u- 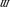 *
3	*pin 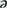 * ‘shrimp’	*pin 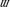 * ‘shrimp species (big, lives in creeks)’	* 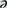 - 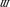 *
4	*agó* ‘bring!’	*a ηe* ‘bring!’ (3sg/bring)	*g-η, e-o*
( = 3+4)	*pin 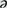 -gó* ‘bring shrimp!’	*pin 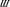 na ηe* (shrimp/3sg/bring) ‘bring shrimp!’	(see 3, 4)
5	*gu* ‘yes’	*η 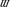 * ‘yes’	*g-η, u- 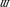 *
6	* 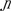 e* ‘no’	* 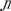 é* ‘emphatic negation’	*e-é*
7	*-nate* ‘father’	*nat 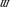 * ‘father’	(exception to *Ø-#n*)
8	*amá* ‘come!’	* 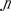 iama* ‘let us follow’	*Ø-# 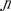 i*
9	*pama* ‘there, look!’	*paamà* ‘Quick!, Hurry up!’	*a-aa*
10	*ao* ‘children to call their father’	*a o* ‘children to call their parents or parents to call children (affective)’	
11	* 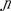 a* ‘out’	*na 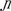 a* (3sg/out) ‘get out!’	
12	*aua* ‘calling a child’	*na u˜ã* (3sg/go) ‘come here!, move!’	*Ø-#n*
13	* 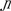 a-nauué* ‘give me, show me’	* 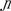 ia na uué* (exhortative/3sg/lower) ‘lower it (e.g., your hand)!’	
14	*hako* ‘bite’	*ja ηo* (exhortative/eat) ‘eat!’; *na ηo* (3sg/eat) ‘he eats’	(*k-η*)
15	* 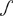 àma* ‘enough!’	*tama* ‘negation’; * 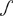 ama* ‘1sg’	
16	*dimene* ‘kill’	*t 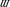 ma˜ni* (1pl/kill/agentive) ‘our killers’; *d 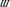 mení* ‘look!’	

Thus the correspondences in Table 7 provide strong indications that Carabayo is genealogically related to Tikuna, but they cannot be taken as evidence for a closer relation with Tikuna than with Yurí, as the larger number of correspondences with Tikuna might suggest. In fact, we may expect a lower number of correspondences with the available Yurí data for a number of reasons. Firstly, Yurí is probably poorly represented, both phonologically and semantically, in the 19th-century data by travelers with no training in linguistics and probably no common language with the Yurí they were interviewing. Secondly, with only a fixed list of Yurí words available, it is naturally much less likely to find matching elements than when a native Tikuna speaker actively searches correspondences to Carabayo items. Indeed, we have initial evidence that Carabayo shares features with Yurí but not with Tikuna, mainly *g* (or *k*) in positions that correspond to Tikuna *
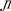
*, e.g. in Yurí *g
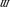
o* – Tikuna *ngoó ˜* ‘snake’ or Yurí *kõ ja* – Tikuna *ngùga* ‘Tinamus bird’. All this suggests that Yurí, Carabayo and the various dialects of Tikuna are genealogically related, with Carabayo somewhere in the middle between Yurí and Tikuna, but probably closer to Yurí. The ease with which Carabayo data could be interpreted by a native Tikuna speaker additionally suggests that these languages are relatively closely related and may even form – or have formed in the past – a dialect continuum.

## Conclusions

This paper presents evidence suggesting that the Carabayo people, who live in voluntary isolation in the Colombian Amazon region, speak a language related to Yurí and also Tikuna, i.e. that they are – direct or indirect – descendants of the Yurís that travelers such as Martius, Spix, Wallace, and Natterer encountered in the 19th century. We were able to provide correspondences to almost all Carabayo items for which reasonably reliable glosses are available. The correspondences we find between Carabayo and Yurí, on the one hand, and Carabayo and Tikuna, on the other hand, are unlikely to be instances of borrowing from Yurí and Tikuna and thus likely to truly reflect a genealogical link.

With the accelerating loss of indigenous languages, it becomes increasingly difficult to gain any further knowledge of the pre-colonial linguistic landscape of Amazonia. However, our meticulous study of Carabayo data from 1969 contributes to putting one language, Carabayo(-Yurí), (back) on the map, and to placing another language, Tikuna, (back) in a linguistic family, Tikuna-Yurí, of which it had been presumed to be the only surviving member. We hope that this study will also contribute to awareness of the existence of groups that avoid contact and especially of their right to be left in peace.
